# Psychometric Properties of the German Version of the Perception of Interprofessional Collaboration Model-Questionnaire (PINCOM-Q)

**DOI:** 10.5334/ijic.5660

**Published:** 2021-10-27

**Authors:** Astrid Jörns-Presentati, Gunter Groen, Atle Ødegård

**Affiliations:** 1 Faculty of Business and Social Sciences, University of Applied Sciences, Hamburg, Germany; 2 Faculty of Health Sciences and Social Care, Molde University College, Specialized University of Logistics, Norway; 3 Nordland Research Institute, Norway

**Keywords:** child welfare, child and adolescent psychiatry, interprofessional collaboration, validation, measurement

## Abstract

**Objective:**

The Perception of Interprofessional Collaboration-Model Questionnaire (PINCOM-Q) measures professionals’ perceptions of interprofessional collaboration in the field of child and youth mental health. The aim of this study was to validate the PINCOM-Q in a sample of German child welfare and child and youth mental health professionals.

**Methods:**

The PINCOM-Q was translated into German and its underlying factor structure was examined using exploratory and confirmatory factor analysis.

**Results and discussion:**

Findings from this study suggest four factors (Interprofessional Climate, Conflict, Role Expectancy and Shared Goals, and Motivation) capture the concept of perceptions of interprofessional collaboration between child welfare and child and adolescent psychiatry.

**Conclusion:**

The use of PINCOM-Q (German) can be recommended as a research tool, investigating professional groups working with children and young people with multiple and complex needs.

## Introduction

Interprofessional collaboration (IPC), defined as ‘‘the process in which different professional groups work together to positively impact health care” [1], is a common and promising approach in the field of child and youth mental health [2]. Studies show that the beneficial effects of psychotherapy on youth mental health are moderate in magnitude and only relatively durable [3]. Other services geared to addressing the multitude of risk factors that contribute to mental health problems [4] are often also needed in the treatment process, including child and youth welfare services. It is estimated that about 50% of children and young people in the child welfare system and 75% of children living in residential care have complex mental health issues [5]. Gaps in mental health care provision have been reported in particular for the residential care population, who tend to exhibit even more severe emotional and behavioural problems [6,7]. Interagency collaboration between child welfare and mental health services has been shown to reduce care fragmentation, improve mental health outcomes, and increase service user engagement and satisfaction [8,9]. Increasing attention is therefore placed on forging interprofessional collaboration between the different professional groups working with children and young people with complex mental health needs who also receive support from welfare services. However, as research on IPC is in a relatively early stage of development, there is a need for measurement tools that can generate empirical evidence on a larger scale to inform the international practice and policy debate about integrated care for children with multiple and complex needs [10,11,12].

**Figure 1:**
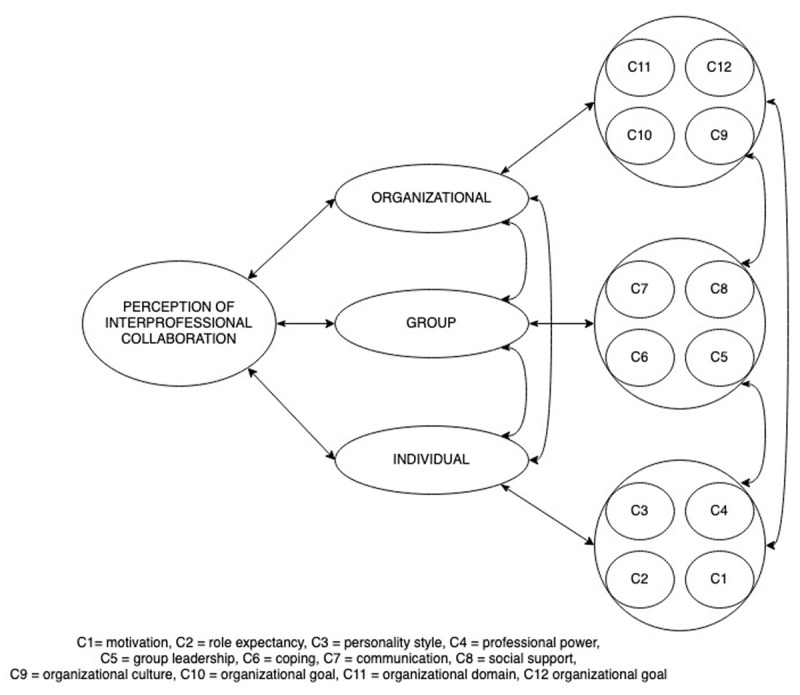
Perception of Interprofessional Collaboration Model [14].

The Perception of Interprofessional Collaboration Model-Questionnaire (PINCOM-Q) is a self-measurement tool developed by Ødegård [13,14] with the purpose of measuring professionals’ perceptions of IPC in the field of child and youth mental health. The underlying assumption of the instrument is that professionals are attuned to key aspects of IPC most salient to them based on their experiences. It is suggested that PINCOM-Q can contribute to exploring shared perceptions or differences in perceptions among professionals in interprofessional groups and/or changes of perceptions over time [14,15]. The scale consists of three dimensions, related to the individual, the group, and the organization. Each of these dimensions is respectively associated with four constructs (see ***Figure 1***). The PINCOM-Q conceptualizes IPC as a broad concept and measures antecedents and processes. Two studies have examined the construct validity of the PINCOM-Q. Ødegård [14] used the scale with a sample of 134 professionals working in primary care, specialist mental health services and elementary schools and arrived at a revised 28-item scale with a six-factor structure. Strype et al. [16] used only items related to perceptions at the individual and at the group level with a sample of 157 members of local youth crime prevention committees and developed an 18-item scale with a three-factor structure. In both studies, the largest subscale (Interprofessional Climate/Group Climate) was composed of items describing social support and communication, which have both been shown to be central aspects of teamwork [17].

### Aims

The main aim of the current study was to assess the underlying factor structure of the PINCOM-Q in a sample of professionals working in child welfare and child and adolescent psychiatry (CAP) in Germany. To our knowledge, no German version of the PINCOM-Q exists to this date. The specific aims were to a) translate the instrument into German and b) examine psychometric properties of the German version of the PINCOM-Q, using exploratory factor analysis (EFA) and confirmatory factor analysis (CFA). A culturally validated version of the PINCOM-Q could serve as a valuable contribution to the research field.

## Method

### Instrument

The development of PINCOM-Q was partly based on the results of a qualitative research study examining professionals’ perceptions of IPC in the child and youth mental health field in Western Norway [13,14]. The scale further builds theoretically on the basic tenants of attribution theory and organisational psychology and measures perceptions of individual, group, and organisational aspects of IPC. At the individual level items represent the constructs personal motivation, role expectations, personality style, and professional power. The constructs at the group level are leadership, communication, coping and social support. At the organizational level the constructs are organizational culture, organizational aims, organizational domain, and organizational environment. Each construct consists of four items, in the following referred to as a, b, c, d. The questionnaire consists of 48 items and statements are rated on a seven-point Likert scale, ranging from strongly agree to strongly disagree. PINCOM-Q has been employed in different settings with a number of different professions and shows high external validity [15,18,19]. A recent systematic review of IPC measurement tools for interprofessional health and social care teams demonstrated the usefulness of the PINCOM-Q with regard to its psychometric properties and usefulness in the field of children’s services [20].

Data for the present study were collected within a larger mixed-methods study [21], examining the impact of a practice-based intervention on collaboration between child welfare and CAP in Northern Germany. A subsection of demographic questions inquiring about the participant’s professional background and years of work experience was included, along with a 12-item perception of interprofessional collaboration scale for children with complex mental health and social care needs, which we developed based on PINCOM-Q. This scale has not been validated yet.

### Translation and cultural adaption process

Permission to use the PINCOM-Q was obtained from the originator. The cultural adaption process followed established guidelines [22]. Two independent forward translations of the original English version of PINCOM-Q into German were carried out by a native-English speaking professional translator with no relevant knowledge about IPC and a first language German speaker with bilingual proficiency and experience in the mental health and social care field. Issues arising in the translation process were discussed and resolved with the first and second author. A German native speaker professionally translated the final German version back into English. All translations were subsequently submitted to a panel consisting of senior faculty members specialized in child welfare, experienced child mental health practitioners, and the first and second author. Conceptual equivalence of each item was again examined and discussed.

It was agreed between the translation and expert panel to retain all the 48 items of the original PINCOM-Q. One item was rephrased: “Some professionals supply the premises in interprofessional groups” on the subscale Professional Power was slightly modified to “Some professionals determine the underlying assumptions in interprofessional groups” to avoid ambiguity in the meaning of the word “premises”. The word “plan” used in two of the four items describing the construct Organizational Aims (“Interprofessional collaboration is well described in their plans” and “I am familiar with the plans of the other organizations”) was not translated literally (Plan), but we used the word “Leitbild” to refer to the overall concept underlying collaborative care planning, which is semantically closer to “mission statement”. Thirteen items were reverse coded, and all items were placed in random order on the questionnaire.

### Data collection

Paper and pencil version of the PINCOM-Q were distributed at project meetings and sent by post to two youth welfare offices, two public child and youth welfare agencies, and to one child and adolescent psychiatric hospital. The study employed a semi-experimental design and therefore a second convenience sample of participant data was collected in equivalent institutions in a neighbouring municipality. The purpose of the study was explained on the first page of the questionnaire and participants were asked to evaluate the interprofessional collaboration between child welfare and CAP based on their experiences. Participation was voluntary and all study participants gave written consent. Ethical approval was attained from all partaking institutions.

### Missing values

Items with the highest percentage of missing values were “Interprofessional collaboration is well described in their plans” (16.1%, n = 56), “Interprofessional work is an area of priority in the other organizations” (10.8%, n = 39), and “The other services have definite and clear aims regarding interprofessional collaboration” (7.5%, n = 27). Missing data for these three items was equally distributed between both cohorts and across professional backgrounds. All three items belonged to the construct Organizational Aims, which was excluded for further analysis. The average percentage of missing values for the remaining 44 items was 1.14% and the responses appeared to be missing completely at random.

### Statistical analyses

The Statistical Package for Social Sciences [23] software was used to calculate descriptive statistics, internal consistency, and to carry out exploratory factor analysis. We used Cronbach’s alpha coefficients > .70 as a cut-off for an acceptable level of reliability [24] and excluded subscales with scores < .50 from factor analysis. The alpha coefficients of the subscales Motivation, Role Expectancy, Personal Style, Professional Power, Coping, Communication, Social Support, Organizational Culture, and Organizational Domain ranged between .55 and .72, indicating modest to good reliability. The subscales Group Leadership (α = .22) and Organizational Environment (α = .20) showed low reliability and were excluded. The relevance of the construct Group Leadership may have suffered due to the lack of a proximal work group [25] in the collaborative practice between CAP and child welfare services in our sample, where IPC is rather consultative in nature. We retained 36 parameters for analysis and therefore fulfilled the suggested 5 observations per item, and the minimum of 100 observations in total [26].

To determine if our material was appropriate for factor analysis, we used the Kaiser-Meyer-Olkin Measure of Sampling Adequacy [27] with a cut off of ≥ 0.5. Our method of extracting factors was principal axis factoring (PAF), as our aim was to explore the underlying structure of the PINCOM-Q in a German sample [28]. Factor retention decisions were based on Kaiser’s criterion that eigenvalues be >1.0 (Nunnally, 1978), and an examination of the scree plot [29]. We retained only factors with no less than three items [30]. Items with loadings weaker than .40 and items that loaded ≥ .40 simultaneously on more than one factor were removed one by one. Confirmatory factor analysis was carried out using lavaan version 0.6–6 [31] in R version 4.0.2 [31]. We used maximum likelihood estimation with robust standard errors and a Satorra-Bentler scaled test statistic (MLM). Model fit was determined with the Comparative Fit Index (CFI), the Tucker Lewis Index (TLI), the Root Mean Square Error of Approximation (RMSEA), the Akaike’s information criterion (AIC), the Bayesian information criterion (BIC), and the Standardized Root Mean Residual (SRMR). For the CFI and TLI, 0.90 is considered the cut off for an adequate and ≥ 0.95 for a close fit of the model [32]. In comparison, a model with a lower score for the AIC and BIC is the preferred model. For the RSMEA a value of ≤ 0.05 was considered a close fit and for the SRMR a value of ≤ 0.08 indicated an acceptable model [32].

## Results

### Participant characteristics

Of the 360 respondents, 64.1% were social workers or child welfare workers, of which around 90% were employed in the child welfare sector. Mental health professionals (nurses, psychologists, psychotherapists, and psychiatrists) made up 27.5% of the entire sample and all of them were employed in the child and adolescent psychiatric hospital. Sixty-three percent of all participants had more than 10 years of experience, and 28% more than 20 years of relevant professional experience. ***Table 1*** shows detailed characteristics of the two study samples.

**Table 1:** Participant Characteristics.


			COHORT 1 (N = 201)		COHORT 2 (N = 159)	
	
			N	%	N	%

Gender						

	Female		149	74.1	110	69.6

	Male		48	23.9	48	30.4

Age						

	under 25		3	1.5	4	2.5

	25–34		53	26.6	41	25.8

	35–44		53	26.6	44	27.7

	45–54		55	27.6	44	27.7

	55–64		34	17.1	25	15.7

	>64		1	0.5	1	0.6

Organisation						

Child and adolescent psychiatry			84	42.2	68	43.0

Child and youth welfare office			82	41.2	48	30.4

Child and youth welfare services			21	10.6	26	16.5

Other			12	6.0	16	10.1

Profession						

Social worker			97	50.0	70	44.3

Child welfare worker			31	16.0	33	20.9

Nurse			19	9.8	14	8.9

Psychologist			6	3.1	4	2.5

Psychotherapist			19	9.8	16	10.1

Psychiatrist			12	6.2	9	5.7

B.A./M.A. Education			4	2.1	2	1.3

Support worker			6	3.1	10	6.3

Work experience						

		under 5 years	41	21.1	31	19.7

		5–9 years	36	18.6	32	20.4

		10–20 years	70	36.1	57	36.3

		>20 years	54	24.2	47	23.6


### Exploratory factor analysis

Thirty-six of the original 48 items of PINCOM-Q were subjected to an exploratory factor analysis. The adequacy of our sample was verified (KMO = .849) and a significant Bartlett’s test of sphericity X^2^ (630) = 2369.56, p < .01 indicated that data were suitable for factor analysis. The initial examination of the eigenvalues and the scree test indicated a four, five or six factor solution. A clean six-factor structure emerged after the removal of eleven items with loadings weaker than .40 and one cross-loaded item (“Everybody knows the area of responsibility of the other professionals”). Factor 6 consisted only of two items “Some professionals lack openness and do not participate much in interprofessional groups” and “Interprofessional collaboration calls for an openness of mind and not all professionals are able to live up to that” of the PINCOM-Q subscale personality style. Both items had high loadings (.81 and 83.), and factor six showed a low but acceptable level of reliability (α = .61). We extracted a five-factor model to see if these two items would group to one of the other five components and the requirement of at least three items loading above .40 would be fulfilled. This model explained 58.48% of the variance in the data and is shown in ***Table 2***.

**Table 2:** Exploratory factor analysis of a five-factor model of the German PINCOM-Q.


CRONBACH’S ALPHA COEFFICIENT		.81	.79	.74	.60	.53	CRONBACH’S ALPHA IF ITEM DELETED

SUBSCALE/ITEM		FACTOR 1	FACTOR 2	FACTOR 3	FACTOR 4	FACTOR 5	

1	Social Support a	**.71**					.78

2	Social support b	**.67**					.78

3	Social support c	**.75**					.77

4	Social support d	**.60**					.80

5	Communication a	**.72**					.78

6	Communication d	**.67**					.79

7	Coping a	**.67**					.78

8	Professional power a		**.81**				.73

9	Professional power b		**.68**				74

10	Professional power c		.59				80

11	Professional power d		**.76**				.76

12	Personality style a		**.76**				.75

13	Personality style d		**.64**				.78

14	Role expectancy c			**.82**			68

15	Role expectancy d			**.73**			.63

16	Organization domain b			**.72**			.62

17	Coping c			**.63**			.69

18	Communication b			**.60**			.72

19	Organizational culture a				**.82**		.57

20	Organizational cultured				**.64**		.59

21	Organization domain a				**.61**		.40

22	Organization domain d				**–59**		.51

23	Motivation a					**.75**	.37

24	Motivation b					**.66**	.48

25	Motivation d					**.61**	.45

26	Role expectancy a					**.55**	.51

	(Cumulative Contribution (%)	27.38%	40.31%	47.60%	53.48%	58.48%	


Factor 1 explained 27.38% percent of the variance and was composed of seven items with loadings ranging between .60 and .75. It was composed of the original subscale Social Support, with two items related to Communication and one item to Coping. The item with the highest loading was “I find that I am appreciated by other professionals in the interprofessional groups I participate in”. Factor 2 explained 12.93% of the variance and item loadings ranged between .64 and .81. Three items derived from the subscales Professional Power and two from Personality Style, with the highest loading item being “Some professionals act in ways that make interprofessional collaboration difficult”.

Factor 3 explained 7.29% of the variance and loadings ranged between .59 to .82. Factor 3 was composed of five items, of which three pertained to Role Expectation, one to Organizational Domain, and one to the construct Communication. The item with the highest loading was “In most of the interprofessional groups I participate in, we agree about priorities” (Coping). Factor 4 consisted of four items and explained a variance of 5.88%, with item loadings ranging between .59 and .81. The two items with the highest loadings were “The organizations are characterized by the wish to work interprofessionally” (Organisation Culture) and “We (employees) are encouraged to promote new ways of working in interprofessional groups” (Organizational Culture). Two items related to the importance of knowing relevant laws, regulations, and responsibilities (Organizational Domain). The smallest factor explained a variance of 5.0% and contained three items of the subscale Motivation and one item of the subscale Role Expectancy (“I always have clear goals when I work interprofessionally”). Item loadings ranged between .55 and .75 and the highest loaded item was “I find working in interprofessional groups valuable”.

### Confirmatory factor analysis and internal consistency

After conducting exploratory factor analysis, we calculated Cronbach’s alpha for each of the five subscales of the PINCOM-Q (German). The cut off for good reliability (≥0.70) was met by three factors. Factor 1 Interprofessional Climate had a Cronbach’s alpha coefficient of 0.81, and factor 3 Role expectancy and Shared Goals had a value of 0.76. Deleting the item “Sometimes I am not able to present my perspectives because other high status professionals talk all the time” increased the value for factor 2 to .80. Cronbach’s alphas for factor 4 Organizational Culture (α = .60) and factor 5 Motivation (α = .53) indicated modest reliability.

**Figure 2:**
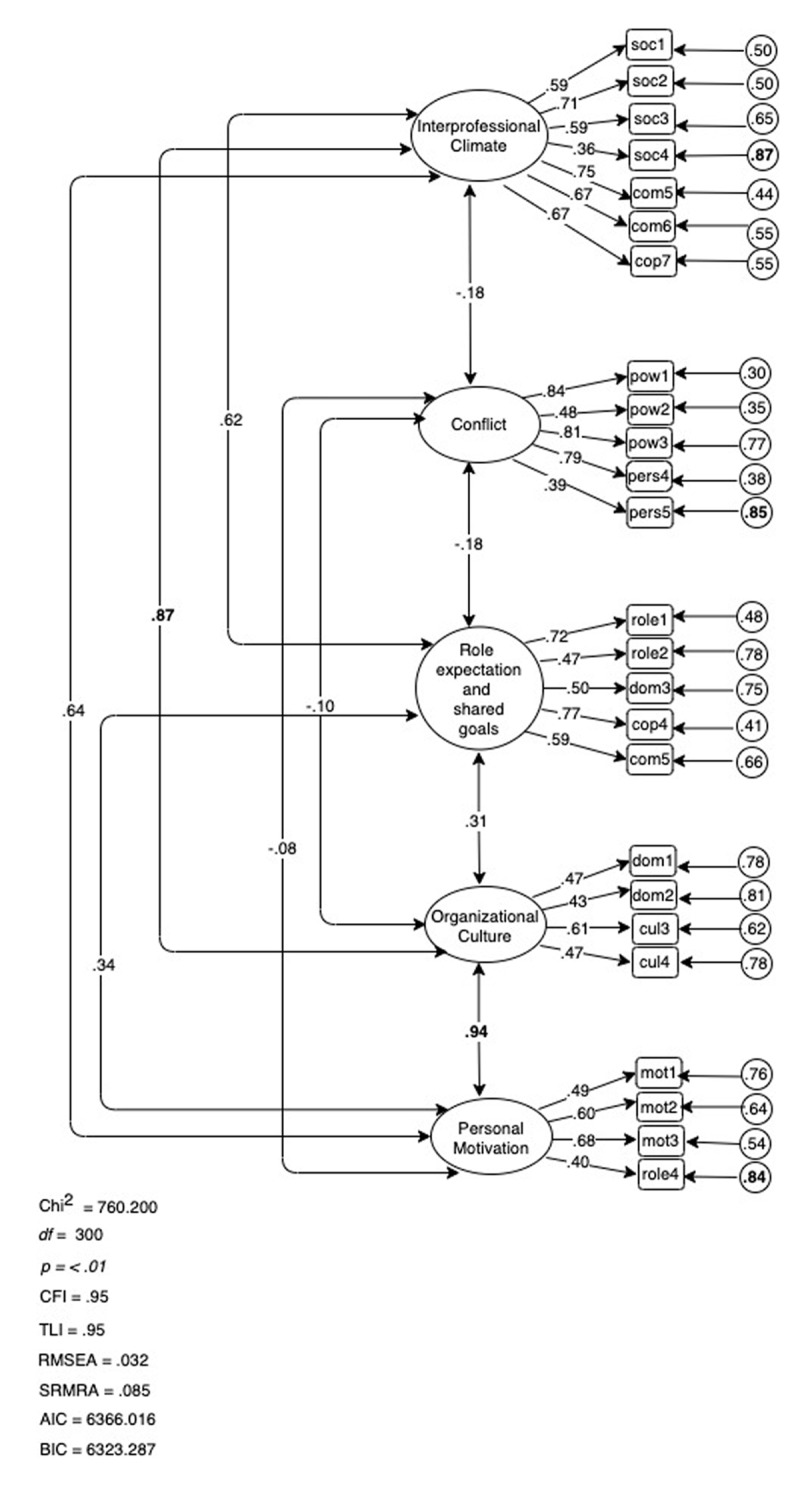
Five-Factor Model of the PINCOM-Q (G).

We subjected the final 25-item five-factor model to a strict confirmatory factor analysis, without allowing any correlated errors in our modelling. The model fit indices nearly fulfilled all criteria for good model fit: χ2 (300) = 760.200, p < .01; RMSEA = 0.032 (90% CI, 0.032; 0.058); CFI = 0.95; TLI = 0.95; SRMR = 0.085; AIC = 6366.016; BIC = 6323.287. As shown in ***Figure 2***, loadings ranged between .36 and .84, which is acceptably high. Factor 4 Organizational Culture showed a very high covariance with Factor 1 Interprofessional Climate (*r* = .87, p < .0001) and Factor 5 Motivation (*r* = .94, p < .0001), indicating that the constructs exhibited insufficient discriminant validity [33].

**Figure 3:**
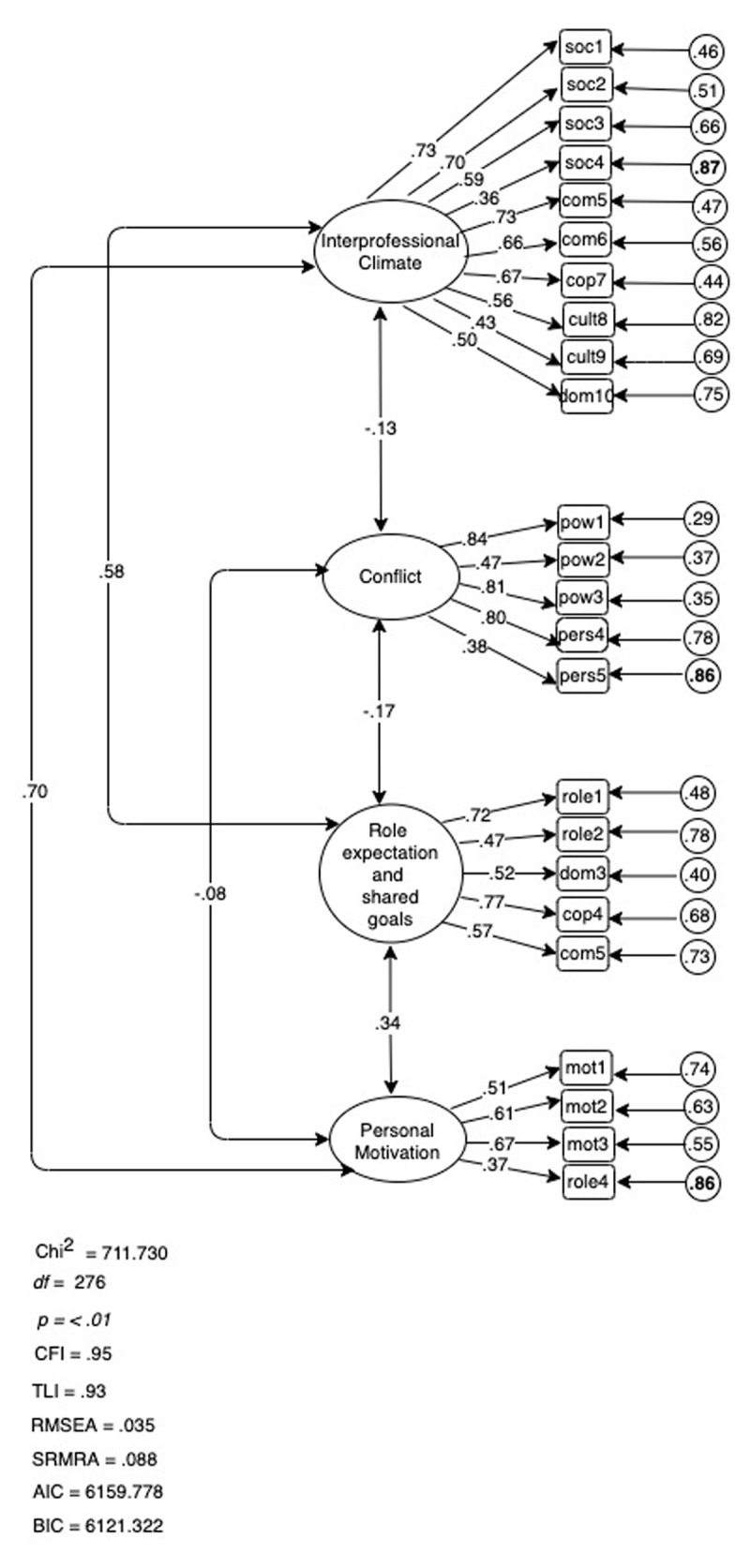
Four-Factor Model PINCOM-Q (G).

Modification indices suggested moving item 19 “The organizations are characterized by the wish to work interprofessionally”, and item 20 “We (the employees) are encouraged to promote new ways of working in interprofessional groups” from factor 5 Organizational Culture to factor 1 Interprofessional Climate. We deemed merging factor 5 and factor 1 theoretically justifiable and tested an alternative four-factor model. As shown in ***Figure 3***, factor 1 Interprofessional Climate consisted in this model of ten items. We added item 19, 20 and 21 (“Laws and regulations are relatively well known by all the professionals in interprofessional groups”) but dropped item 43 (“We need to inform each other about our area of responsibility”), as this increased Cronbach’s alpha to .81. The final overall Cronbach alpha was .85, showing good internal consistency of the PINCOM-Q (G).

## Discussion

In this study, we present psychometric properties of the first German translation of the Perception of Interprofessional Collaboration-Questionnaire (PINCOM-Q). We arrived at our version of the PINCOM-Q (German), as shown in ***Table 3***, following EFA and CFA using a sample of professionals collaborating at the intersection of child welfare and CAP. Our initial results suggested a five-factor structure, but due to high covariance between factors we merged factor 4 and factor 1 to yield a four-factor structure, which was eventually confirmed to be the best fit for our data. The PINCOM-Q (G) therefore shows very promising results in regard to measuring perceptions of interprofessional collaboration in a German-speaking sample.

**Table 3:** PINCOM-Q (G) Items and Subscales.


		CRONBACH’S ALPHA

**Subscale 1**	**Interprofessional climate**	α = .81

1	I experience that I can get help and social support from the other professionals in the interprofessional groups I participate in	

2	I find that other professionals in the interprofessional collaboration groups I participate in, arc willing to listen to me if I have problems	

3	I find that I am appreciated by other professionals in the interprofessional groups I participate in	

4	I have almost never found that other professionals do not understand what I am trying to express and/or report	

5	I get relevant feedback on my contributions in the interprofessional groups I participate in	

6	Professionals arc good at exchanging information with each other about how they work	

7	We almost always solve the defined problems in the interprofessional group	

8	The organizations are characterized by the wish to work interprofessionally	

9	We (the employees) are encouraged to promote new ways of working in interprofessional groups	

10	Laws and regulations arc relatively well known by all the professionals in interprofessional groups	

**Subsacle 2**	**Conflict**	α = .80

1	Some professionals dominate the interprofessional meetings with their professional viewpoints*	

2	Some professionals determine the underlying assumptions in interprofessional groups	

3	Occasionally interprofessional groups do not work because some professionals dominate the meetings*	

4	Some professionals act in ways that make interprofessional collaboration difficult*	

5	Interprofessional collaboration calls for openness of mind and not all professionals are able to live up to that*	

**Subscale 3**	**Role expectancy and Shared Goals**	α = .76

1	My experience is that our roles are always clearly defined	

2	I experience that my area of responsibility is clearly defined when I work in interprofessional groups	

3	Everybody knows their area of responsibility	

4	In most of the interprofessional groups I participate in, we agree about priorities	

5	In the interprofessional groups I participate in, exchange of information is never a problem	

**Subscale 4**	**Motivation**	α = .53

1	I find working in interprofessional groups valuable	

2	I get to use my creativity and imagination when I work in interprofessional groups	

3	I experience personal growth when I work in interprofessional groups	

4	I always have clear goals when I work interprofessionally	

Total: 24 items		α = .85


* Reverse coded.

However, a few limitations apply. Discriminant and convergent validity are core elements of construct validation and should be investigated in future studies. Furthermore, in our analysis we reduced the original 48 items of the PINCOM-Q quite drastically to 24 items. This may have had a negative effect on content validity. Prior validation studies of the PINCOM-Q [14,16] arrived at similarly trimmed versions and we therefore consider this acceptable, in terms of parsimony. This is the first attempt of a German translation of PINCOM-Q and we recommend that future studies undertake further steps of cross-cultural validation, such as conducting individual cognitive interviews with professionals that focus on evaluating each item thoroughly in regard to comprehensibility, conceptual equivalence, and practical usefulness.

The PINCOM-Q (G) showed overall satisfying internal consistency with a Cronbach’s alpha coefficient of .85. Coefficient scores for the subscales were above the recommended .70 cut off [30] for factor 1 (𝛼 = .81), factor 2 (𝛼 = .80) and factor 3 (𝛼 = .76), while factor 4 showed rather low reliability (𝛼 = .53). The results of our study are further indication that PINCOM-Q (G) is a reasonably valid and reliable tool to measure perceptions of IPC in the field of child and youth mental health. However, it is worth noting that reliability is dependent on context and not an inherent quality of the measurement [34]. We used a small and possibly not entirely representative convenience sample, however, it was justified for use with an exploratory and confirmatory analysis.

### Interprofessional climate

Our largest subscale replicates the social support/communication items of the Interprofessional/Group Climate dimension reported in previous validation studies [14,16]. In our study four additional items describing perceived effectiveness, organizational support and mutual awareness of systemic determinants of the specific IPC context also loaded on factor 1. This finding is unique as it captures organizational and group dimensional aspects in the construct underlying the dimension Interprofessional Climate. A strong correlation between the organizational dimension and the group dimension of the PINCOM-Q has been reported before in a sample of youth mental health professionals in Canada [18] and organisational support has been found to be associated with specialized mental health teams [35]. From a conceptual point of view, it is reasonable to think that organizational support is critical for the interprofessional climate in working with children and young people with complex needs. IPC between child welfare and CAP frequently occurs in extreme situations, for example, if a child experiences serious family conflicts involving child protection and mental health services or a young person living in a residential group home is in a severe crisis that requires safety monitoring and psychiatric assessment. Professionals therefore often have to make decisions on the fly due to a high degree of urgency, complexity and unpredictability [36] of problems and need a strong organisational back up.

### Conflict

We labelled our second subscale “Conflict” as it describes the lack of openness and professional power as two different causes for contention in IPC. There is substantial evidence in the literature that IPC between social workers and medical professionals can be perceived as conflictual due to the fact that social work has traditionally been assigned the role of an ancillary profession to psychiatry [11,37]. Unrelated to professional power, conflict may arise if professionals have negative perceptions about each other due to mistrust, stereotypes or low expectations as shown by Widmark et al. [38] in their study of social representations of IPC in the welfare sector. Results of our initial EFA indicated that a lack of openness resonated strongly with professionals in our sample and it may therefore be worth generating more items describing openness to different perspectives or openness to change, which have been shown to be relevant for IPC in general [39], and in particular with regard to young people in residential care [40].

### Role expectation and shared goals

The third subscale is unique to our validation study and we labelled it “Role Expectation and Shared Goals”. Interprofessional working relationships benefit from a clear distribution of roles and responsibilities among professionals with strong professional identities [41]. However, the items of this subscale relate to both, professional identification (“My experience is our roles are always clearly defined”) and team identification (“In most of the interprofessional groups I participate in, we agree about the priorities”). Kebe et al. (2020) refer to this as multifocal identification, which they found to be a factor associated with IPC in specialized mental health care teams and primary mental health care teams in their study [35]. Furthermore, professionals considered the successful exchange of information important in the division of responsibilities, suggesting that knowledge integration, a concept that “involves the development of a relational dynamic in which professionals influence each other in analysing the situations encountered, and in articulating a shared vision and plan of action” [35] is a relevant component of developing shared goals.

### Motivation

The fourth factor labelled “Motivation” was reported in both prior validation studies [14,16] and replicates items of the original construct, and one additional item “I always have clear goals when I work interprofessionally”. This makes conceptual sense as it has been shown that IPC can have negative impacts, such as an increased workload [2] or putting professionals under additional stress [42]. A clear vision of the potential benefits can increase motivation, commitment, and willingness to collaborate.

### Implications for practice and future research

Our goal was to provide a useful tool to German speakers investigating perceptions of IPC at the intersection of the child welfare and child mental health system. Given that previous research has shown that professionals working at the intersection of child welfare and child mental health face similar challenges across cultures [11], we assume that our study also has merit for the international community. The psychometric properties of the German version of the PINCOM-Q are comparable to the English language version of the scale. As the original version of the scale has only been validated in a Norwegian context, future validation studies of the PINCOM-Q should use English-speaking samples to add to the conceptualization of IPC as a construct.

Our data suggests that professionals perceived the relational quality of IPC to be the central element of their collaborative practice. This finding is in line with recent international qualitative research showing that professionals working with children with complex needs across settings and disciplinary background emphasize the importance of mutual understanding, shared thinking, and familiarity with other professionals [2,10,43]. Further studies should therefore build on our results and use the scale to measure changes of perceptions of IPC over time that may indicate effects of longer-term practice-based IPC interventions, such as interprofessional education or joint care planning.

To further the conceptual clarity of the Interprofessional Climate dimension of the PINCOM-Q (G), we suggest that future studies examine the relationship between the PINCOM-Q and the Team Climate Inventory developed by Anderson and West [25] to see if there is an underlying connection with the construct “Participative Safety”. The emphasis on a positive work environment as a necessary condition for shared decision-making seems relevant as Rousseau et al. [18] reported a moderate correlation between the PINCOM-Q and an adapted version of the Decisional Conflict Scale. This may indicate that creating an atmosphere that allows shared-decision making is an aspect of the construct Interprofessional Climate and a component of the nomological network surrounding the PINCOM-Q [44].

## Conclusion

This study contributes to the interprofessional measurement literature by adding to the construct validation process of a tool that has already been established. Findings from this study suggest a four-factor structure of the PINCOM-Q (G) and the translation into German was successful. The tool can therefore be considered a valid and reliable instrument for assessing professionals’ perceptions of IPC with children and young people with complex needs.
